# Detection of Long Non-Coding RNA in Archival Tissue: Correlation with Polycomb Protein Expression in Primary and Metastatic Breast Carcinoma

**DOI:** 10.1371/journal.pone.0047998

**Published:** 2012-10-25

**Authors:** Karen M. Chisholm, Yue Wan, Rui Li, Kelli D. Montgomery, Howard Y. Chang, Robert B. West

**Affiliations:** 1 Department of Pathology, Stanford University School of Medicine, Stanford, California, United States of America; 2 Howard Hughes Medical Institute and Program in Epithelial Biology, Stanford University School of Medicine, Stanford, California, United States of America; The Institute of Cancer, Research-London, United Kingdom

## Abstract

A major function of long non-coding RNAs (lncRNAs) is regulating gene expression through changes in chromatin state. Experimental evidence suggests that in cancer, they can influence Polycomb Repressive Complexes (PRC) to retarget to an occupancy pattern resembling that of the embryonic state. We have previously demonstrated that the expression level of lncRNA in the *HOX* locus, including *HOTAIR*, is a predictor of breast cancer metastasis. In this current project, RNA in situ hybridization of probes to three different lncRNAs (*HOTAIR*, nc*HoxA1*, and nc*HoxD4*), as well a immunohistochemical staining of EZH2, is undertaken in formalin-fixed paraffin-embedded breast cancer tissues in a high throughput tissue microarray format to correlate expression with clinicopathologic features. Though overall EZH2 and *HOTAIR* expression levels were highly correlated, the subset of cases with strong *HOTAIR* expression correlated with ER and PR positivity, while the subset of cases with strong EZH2 expression correlated with an increased proliferation rate, ER and PR negativity, HER2 underexpression, and triple negativity. Co-expression of *HOTAIR* and EZH2 trended with a worse outcome. In matched primary and metastatic cancers, both *HOTAIR* and EZH2 had increased expression in the metastatic carcinomas. This is the first study to show that RNA in situ hybridization of formalin fixed paraffin-embedded clinical material can be used to measure levels of long non-coding RNAs. This approach offers a method to make observations on lncRNAs that may influence the cancer epigenome in a tissue-based technique.

## Introduction

One aspect of tumor development involves the alteration of gene expression patterns due to epigenetic changes. The Polycomb group (PcG) proteins work in multiprotein complexes called Polycomb Repressive Complexes (PRCs) that repress transcription of gene expression by modification of chromatin. PcG proteins bind and repress promoters of genes that encode proteins with key roles in cell fate determination and in embryonic development. During cell fate determination, PcG proteins are displaced and recruited to different subsets of target genes. In cancer, PcG target genes are frequently epigenetically silenced by DNA methylation [1]–[3]. This silencing may be due to the high expression of PcG proteins in cancer [4].

EZH2, the human homolog of the *Drosophila* protein Enhancer of Zeste, is a PcG protein in the PRC2 complex [5]. EZH2 is amplified and highly expressed in many cancers including melanoma, endometrial, prostate, and breast carcinoma [6]–[10]. In breast carcinoma, EZH2 protein levels have been found to be strongly associated with poor clinical outcomes [8]. Kleer *et al*. [8] identified EZH2 to be overexpressed in invasive and metastatic breast cancer compared to normal breast epithelial cells. In addition, they found that EZH2 levels significantly correlated with negative ER status, negative PR status, and lymph node status, but not Her2 overexpression. Later, groups found that EZH2 was also significantly associated with tumor cell proliferation [6], [11].

Long non-coding RNA (lncRNA) may be one of the regulators of PcG proteins. LncRNAs are RNAs that are longer than 200 nucleotides in length and do not code for proteins, although they can interact with proteins. They are thought to have diverse functions including imprinting, X chromosome inactivation, chromatin remodeling, transcription regulation, cell cycle control, and possibly be candidate oncogenes and tumor suppressors [12]–[14]. Over 8,100 lncRNAs have been cataloged to date [15]. Experimental evidence suggests that in cancer, they can influence PRCs to retarget to an occupancy pattern resembling that of the embryonic state. Approximately 20% of all human lncRNA have been observed to bind to the PRC2 complex, leading to the proposal that lncRNA guide PcG proteins to their target genes [16].

In a recent paper by Gupta *et al*. [17], lncRNA in the *HOX* loci were found to become dysregulated during breast cancer progression. This study identified a distinct set of *HOX* lncRNA to be overexpressed in primary tumors and very frequently overexpressed in metastases. One such lncRNA, *HOTAIR*, was increased in primary tumors and metastases, and its expression level in primary tumors was a predictor of eventual metastasis and death. *HOTAIR* had previously been shown to recruit PcG proteins to chromatin through interaction with the PRC2 complex [18]. Overexpression of *HOTAIR* induced localization of PRC2 subunit EZH2 onto many genes; this PRC2 occupancy pattern more resembled the embryonic state [17].

In this study, we measured the expression of lncRNAs in formalin-fixed paraffin-embedded (FFPE) tissues by in situ hybridization to understand how lncRNA expression is correlated with clinical features. We use RNA in situ hybridization probes of *HOTAIR* and two other *HOX* locus lncRNAs (nc*HoxA1* and nc*HoxD4*), which were identified in the Gupta *et al*. paper [17] to be co-expressed in metastatic breast carcinomas, to see if *HOX* locus lncRNA expression and EZH2 protein expression correlate with clinicopathologic features. Lastly, using matched primary and metastatic breast carcinomas we determine if *HOTAIR* and EZH2 have increased expression in metastatic versus primary breast carcinoma.

## Materials and Methods

### LncRNA Probes

Probes of 400 to 500 nucleotides were created based upon unique non-conserved sequences and constructed as previously described [17]. In brief, multiple antisense probes targeting different parts of each of the lncRNA sequences were developed based upon predictions of the lncRNA secondary structures. Sequences that had high evolutional conservation were avoided, as they may be preferentially involved in tertiary RNA structures that could be difficult to hybridize to in a FFPE environment. In addition, sense stranded probes (opposite strand to the targeting antisense probe) were constructed for each lncRNA to evaluate for non-specific hybridization. The sense and antisense RNA probes labeled with Digoxigenin (DIG) were generated by PCR amplication of a T7 promotor which was incorporated into the primers. Per manufacturer’s protocol (Roche Diagnostics), a DIG RNA labeling kit and T7 polymerase performed *in vitro* transcription. The primers used to construct these probes are as follows: HOTAIR Anti Sense Forward: gcagtggggaactctgactc, HOTAIR Anti Sense Reverse: CTAATACGACTCACTATAGGGgcttgggtgtaattgctggt, ncHoxA1-53 Anti Sense Forward: agtgctggagcgaagaagag, ncHoxA1-53 Anti Sense Reverse: CTAATACGACTCACTATAGGGgaaaacgcagcatgtaagca, nc-HoxD4-27 Anti Sense Forward: ttgagatgaggttcccaagc, nc-HoxD4-27 Anti Sense Reverse: CTAATACGACTCACTATAGGGgccctcgtctcgtattttca.

### RNA *in situ* Hybridization

The RNA in situ hybridization was performed as previously described [17]. Hybridization included sense or antisense riboprobes at 200 ng/ml dilutions. The stains were then scored by eye by authors (KC and RW), on a two- or three-tiered scoring system, using the following criteria for the two-tiered system: 0 = negative; 1 = equivocal/uninterpretable; 2 = positive; and for the three-tiered system: 0 = negative; 1 = equivocal/uninterpretable; 2 = weak positive; 3 = strong positive.

### EZH2 Antibody

The primary EZH2 antibody used was BD Transduction Laboratories, clone 11, at a 1∶25 titration. The immunohistochemical reactions were visualized using Vector Elite ABC kit (BD Transduction Laboratories). The intensity of staining was interpreted by histopathologic evaluation by the primary author (KC), using the following criteria: 0 = negative; 1 = equivocal/uninterpretable; 2 = weak positive; 3 = strong positive.

### Breast Tissue Microarrays

Breast tumors were collected and studied using Health Insurance Portability and Accountability Act (HIPAA)-compliant Stanford University Medical Center institutional review board (IRB) approval. The Stanford IRB waived the need for written consent from the participants due to the use of archival tissue. This study is reported according to the Recommendations for Tumor Marker Prognostic Studies (REMARK) criteria [19]. Two tissue microarrays comprised of formalin-fixed paraffin embedded tissues were used: the first microarray (TA-221) contained 283 primary breast carcinomas and control specimens [20], [21], and the second microarray (TA-248) contained 110 metastases from the primary breast cancers on the first array as well as control specimens [22]. Of the 110 metastases, 56 different cases were represented (some cases had multiple metastases). For each of the primary breast carcinomas, clinicopathologic features including metastasis, hormone status (ER, PR, and HER2/neu), and Ki67 proliferation index had previously been identified (Table 1). Not all cases had all data available. Statistics were performed using a two-tailed Fisher exact test and Chi-squared test. Serial sections of 4 µm were cut from the tissue microarray block and used for in situ hybridization and immunohistochemical analysis.

**Table 1:** Clinicopathologic features of primary breast carcinoma from breast tissue microarray.

Feature	N
**Metastases**	109/261 (42%)
**ER+**	194/236 (82%)
**PR+**	170/236 (72%)
**Her2>2.2 Her2<1.8**	19/222 (8.6%) 194/222 (87%)
**Triple negative**	29/240 (12%)
**Ki67>10% Ki67≤10%**	134/237 (57%) 103/237 (43%)

Outcome data for the 243 of the 283 breast cancer cases represented in TA-221 was obtained through the Stanford Cancer Registry. The median follow-up for living patients is 1897 days (62 months), with a range of 325 to 3456 days. GraphPad Prism was used to compute Kaplan–Meier survival curves. The log-rank test for trend p-value was calculated to assess the probability that there was a trend in survival scores across the groups. Overall survival (OS) was defined by death from any cause.

## Results

### LncRNA Scoring

**Figure 1:**
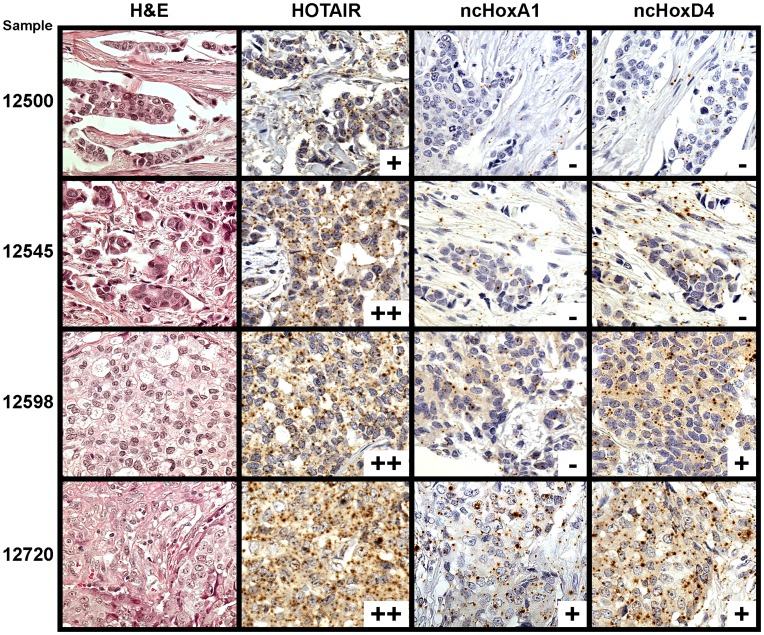
Expression levels in four different breast lesions. Four different breast lesions, in rows, are stained each for hematoxylin and eosin (H&E), and RNA in situ probes for *HOTAIR*, nc*HoxA1*, and nc*HoxD4*, in columns. Expression levels of nc*HoxA1*, and nc*HoxD4* are depicted as negative (−) and positive (+). For *HOTAIR*, expression levels are depicted as negative (−), weakly positive (+), and strongly positive (++).

In order to determine if lncRNA can be measured in formalin-fixed paraffin embedded tissue, probes of 400 to 500 nucleotides based upon unique lncRNA sequences to *HOTAIR*, nc*HoxA1*, and nc*HoxD4* were created. Using RNA in situ hybridization, these probes were hybridized to the breast carcinoma tissue microarrays. Figure 1 depicts representative staining of four breast lesions with these in situ hybridization probes. The RNA probes stained predominantly as dot-like patterns in the cytoplasm surrounding the nucleus, as well as scattered dots within the nucleus. This pattern is similar to previous experiments that we have performed and reported in Gupta *et al*. in which we validated the use of these probes [17] (please see Supplementary Figure 7 in Gupta *et al*. [17]). In that journal article, we transduced *HOTAIR* into breast cancer cell line MDA-MB-231 which allowed stable overexpression of *HOTAIR* greater than 1000 fold. A cell line with no *HOTAIR* expression was also used as a control. These cell lines were implanted into mice and allowed to form lung metastases, which were then formalin-fixed and embedded into paraffin. RNA in situ hybridization of *HOTAIR* in the *HOTAIR* over-expressing cell line demonstrated nuclear and cytoplasmic staining that was specific to only those cells that were transduced; *HOTAIR* was not identified in the surrounding tissues or in the tissues that were transduced with the *HOTAIR* non-expressing cell line.

On the breast carcinoma tissue microarrays, the probes were scored on a two or three-tiered scale: nc*HoxA4* and nc*HoxD1* were scored negative versus positive; *HOTAIR,* which demonstrated a wider dynamic range of staining intensity, was scored negative versus weakly positive versus strongly positive. Of the 283 lesions on the primary breast carcinoma tissue microarray, unequivocal scores were obtained in 221/283 (78%) *HOTAIR*, 118/283 (42%) nc*HoxA1*, and 142/283 (50%) nc*HoxD4* carcinomas. nc*HoxA1* and nc*HoxD4* had the same expression score in the same tissue sample 82.6% of the time (*p*<0.0001). *HOTAIR* and nc*HoxD4* had the same score in the same tissue 68.4% of the time (*p*<0.0004). *HOTAIR* and nc*HoxA1* had the same score in the same tissue 53.1% of the time.

### EZH2 Scoring

EZH2 is a Polycomb group protein known to interact with lncRNA, including *HOTAIR* [17]. In order to determine if all three lncRNA *HOTAIR*, nc*HoxA1*, and nc*HoxD4* co-express with EZH2, and to help determine the roles of both the lncRNA and Polycomb protein in breast carcinoma, EZH2 protein immunohistochemical staining was performed on the breast carcinoma microarrays. The EZH2 antibody staining was scored on a three-tiered scale similar to *HOTAIR*. Of the 283 lesions on the primary breast carcinoma tissue microarray, unequivocal scores were obtained in 218/283 (77%). EZH2 had the same expression score in the same tissue sample as nc*HoxA1* 40.4% of the time, nc*HoxD4* 47.7% of the time, and *HOTAIR* 75.7% the time (*p*<0.0001).

### Clinicopathologic Correlation

Outcomes of breast cancer depend on a number of variables, including the presence of metastasis, hormone receptor status, and proliferation rate. In the breast carcinoma tissue microarray employed, each primary breast carcinoma had known clinicopathologic data including metastasis, ER status, PR status, HER2/neu status, and Ki67 for proliferation index. Though EZH2 levels are known to significantly correlate with negative ER status, negative PR status, lymph node status, and tumor cell proliferation [6], [8], [11], and *HOTAIR* levels correlate with eventual metastasis and death [17], other clinicopathologic correlations between *HOTAIR* and the other two lncRNAs (nc*HoxA1* and nc*HoxD4*) are unknown. Using the known clinicopathologic data from the breast carcinoma microarray, these correlations can be determined. As depicted in Table 2, strongly positive *HOTAIR* expression significantly correlated with ER positivity (*p = *0.026) and PR positivity (*p = *0.0004). Under the usual proliferation rate divisions of ≤10% and >10%, *HOTAIR* did not significantly correlate with proliferation index. nc*HoxA1* did not significantly correlate with any clinical data, but expression was higher with a higher proliferation index (*p = *0.222). Likewise, nc*HoxD4* did not significantly correlate with any clinical data, but had higher expression with PR positivity (*p = *0.288). However, when nc*HoxA1* and nc*HoxD4* had correlating expression levels, the proliferation rate was more often increased (*p = *0.043), especially when they both had increased expression (*p = *0.052). Alone, lncRNA and EZH2 expression levels did not significantly correlate with metastasis.

**Table 2:** Summary of lncRNA and EZH2 associations with clinicopathologic data.

	ER status	PR status	Her2	Triple Negative	Ki67
***HoxA1***	NS	NS	NS	NS	Increased p = 0.222
***HoxD4***	NS	Positive p = 0.288	NS	NS	NS
***HOTAIR***	Positive −/+ vs ++ p = 0.026	Positive −/+ vs ++ p = 0.0004	NS	NS	NS
**EZH2**	Negative p = <0.0001	Negative p = <0.0001	Negative p = 0.004	p = 0.0014	Increased p = <0.0001

NS = not significant.

− = negative.

+ = weak positive.

++ = strong positive.

Strongly positive EZH2 was confirmed to correlate with ER negativity (*p*<0.0001), PR negativity (*p*<0.0001), and proliferation index (*p*<0.0001). In addition, strongly positive EZH2 scores correlated with the lack of Her2 overexpression (*p = *0.004) and triple negative breast carcinomas (*p = *0.0014).

### Primary Versus Metastatic Carcinoma

**Figure 2:**
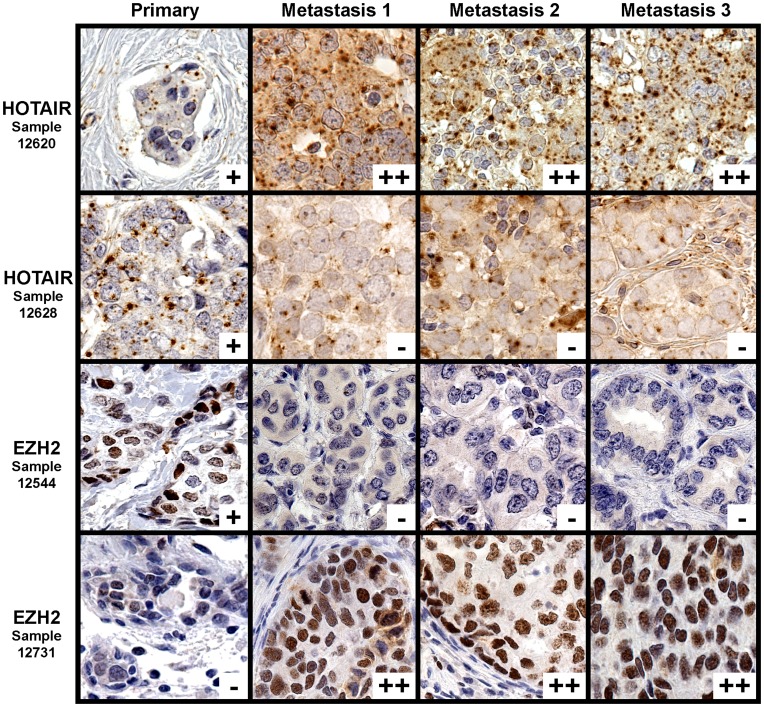
Expression of *HOTAIR* and EZH2 in primary versus metastatic carcinoma. Primary breast carcinomas with their matched metastases are stained with an RNA in situ hybridization probe for *HOTAIR* (rows 1 and 2) or immunohistochemical antibody for EZH2 (rows 3 and 4). Expression levels are indicated as negative (−), weakly positive (+), and strongly positive (++). As depicted, some metastatic carcinomas have increased expression compared to their matched primary carcinomas, while some metastatic carcinomas have decreased expression compared to their matched primary carcinomas.

Though both *HOTAIR* lncRNA and EZH2 protein have been found to be overexpressed in both primary and metastatic breast carcinomas [8], [17], their expression in matched primary and metastatic cancers has yet to be explored. As formalin-fixed paraffin embedded tissue can now be employed to measure lncRNA qualitatively, matched primary and metastatic breast carcinomas can more easily be identified. A metastatic breast carcinoma tissue microarray containing 110 metastatic lesions matched to 56 primary carcinomas on the first breast cancer microarray was used to determine the expression of both *HOTAIR* and EZH2 in the matched pairs. On the metastatic breast carcinoma tissue microarray, unequivocal scores were obtained in 81/110 (74%) *HOTAIR*-stained lesions, and 101/110 (92%) EZH2-stained lesions. For *HOTAIR*, 11 metastases were negative, 48 were weakly positive, and 22 were strongly positive; in reference, on the primary breast carcinoma array, 40 carcinomas were negative, 131 were weakly positive, and 50 were strongly positive. Fifty four (54) pairs were present in which both the primary and the metastatic carcinoma had unequivocal scores. When pairing the primary breast carcinoma with its metastatic focus/foci, there was a significant enrichment for increased *HOTAIR* expression over equivalent or decreased expression, with nine (9) pairs having decreased *HOTAIR* expression at the metastatic focus (17%), 17 pairs having equivalent expression (31%), and 28 pairs having increased expression (52%) (*p = *0.0064). Figure 2, row 1, demonstrates one such carcinoma pair in which *HOTAIR* expression is increased in metastases compared to the primary carcinoma, while row 2 demonstrates a carcinoma with decreased *HOTAIR* expression in the metastases.

For EZH2, 4 metastases were negative, 59 were weakly positive, and 38 were strongly positive; in reference, on the primary breast carcinoma array, 24 carcinomas were negative, 159 were weakly positive, and 35 were strongly positive. Eighty-two (82) pairs were present in which both the primary and the metastatic carcinoma had unequivocal scores. When pairing the primary breast carcinoma with its metastatic focus/foci, there was a significant enrichment for equivalent or increased EZH2 expression versus decreased expression, with six (6) pairs having decreased EZH2 expression at the metastatic focus (7%), 43 pairs having equivalent expression (52%), and 33 pairs having increased expression (40%) (*p*<0.0001). Figure 2, row 4, demonstrates increased EZH2 staining in metastatic foci compared to their matched primary carcinoma, and row 3 demonstrates a carcinoma with decreased EZH2 expression in the metastatic foci.

### Overall Survival

**Figure 3:**
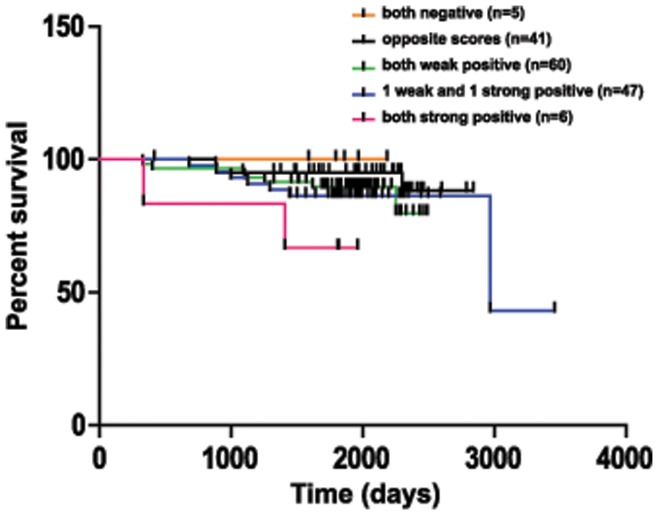
Strong EZH2 and *HOTAIR* **coexpression trend with worse survival.** Kaplan-Meier curve of overall survival based upon the primary carcinoma expressions of both EZH2 and *HOTAIR* stratified into five different groups: both EZH2 and *HOTAIR* negative scores (orange line), one negative and one positive score (black line), both EZH2 and *HOTAIR* weakly positive scores (green line), one weak positive and one strong positive score (blue line), and both EZH2 and *HOTAIR* strongly positive scores (pink line). The number of cases in each group is listed. As the expression of both EZH2 and *HOTAIR* become positive and stronger, the percent survival tends to decrease. Strong expression of both EZH2 and *HOTAIR* together correlate with a trend toward worse survival (logrank test for trend p-value 0.0739).

Gupta *et al*. [17] identified an interdependency between *HOTAIR* and EZH2 in promoting cancer invasiveness. In order to determine if expression of the lncRNA and protein together had an influence on patient outcome, the individual scores on the primary breast cancer tumor array were stratified into five groups as follows: both EZH2 and *HOTAIR* negative scores, one negative and one positive score, both EZH2 and *HOTAIR* weakly positive scores, one weak positive and one strong positive score, and both EZH2 and *HOTAIR* strongly positive scores. As outcome data was available for individuals who were sampled on this tissue microarray, a Kaplan Meier curve was constructed based on these five groups (Figure 3). Of the 243 cases with outcome data, 38 did not survive. Of these 243 cases, both *HOTAIR* and EZH2 expression levels were available in 159 individuals. As illustrated by Figure 3, strong EZH2 expression together with strong *HOTAIR* expression correlates with a trend toward worse survival (logrank test for trend p-value 0.0739), though only six samples are in this group.

## Discussion

### RNA *in situ* Hybridization on Formalin-fixed Paraffin Embedded Tissue

This is the first study to show that RNA in situ hybridization of formalin-fixed paraffin embedded (FFPE) clinical material can be used for qualitative measurements of long non-coding RNAs. In creating the lncRNA probes, multiple difficulties were broached. LncRNAs can derive function from forming secondary structures, including hairpins loops. These secondary structures can sterically hinder the ISH probe hybridization. We were able to anticipate the sequences that form secondary structures through detailed sequence analyses of the lncRNAs including phylogenetic analysis for conserved sequences. We avoided sequences that had high evolutional conservation, as they may be preferentially involved in tertiary RNA structures that could be difficult to hybridize to in a FFPE environment. Using this approach we developed multiple probes that targeted different parts of the lncRNA sequence. In addition, lncRNAs are not only known to have decreased expression levels compared to protein-coding genes, but also to have more tissue specificity in their expression patterns [15], so we had to develop a probe that would be able to identify low expression levels. The specificity of the probe for *HOTAIR* was verified in our prior report [17], in which a breast cancer cell line MDA-MB-231 was transduced with *HOTAIR*. The cell line was implanted into mice and allowed to form lung metastases, which were then fixed in formalin and embedded into paraffin. RNA in situ hybridization of *HOTAIR* in the resulting FFPE tissue demonstrated staining in only those cells that were transduced.

RNA probes for nc*HoxA1*, nc*HoxD4*, and *HOTAIR* were created and found to create a dot-like pattern in the cytoplasm surrounding the nucleus upon in situ hybridization (Figure 1). Interestingly, lncRNA in situ hybridization did not specifically localize in the nucleus, whereas EZH2 immunohistochemistry did. This lncRNA staining pattern is similar to the nuclear and cytoplasmic staining pattern in our previous report in which *HOTAIR* was transduced into breast cancer cell line MDA-MD-231 [17]. Though this current project was not using cell lines, the finding of nuclear and cytoplasmic *HOTAIR* is consistent. Of note, single molecule RNA FISH against *HOTAIR* in primary lung and foot fibroblast cells showed both nuclear and cytoplasmic localization [16], and recent chromatin immunoprecipitation experiments have localized *HOTAIR* in conjunction with PRC2 complex on chromatin in cancer cells [14]. Interestingly, the latter study [14] showed that efficient detection of chromatin-localized RNA required nonreversible glutaraldehyde crosslinking, but was lost with reversible formaldehyde crosslinking. Thus, the current FFPE in situ hybridization experiments may have insufficient resolution or efficiency to strongly detect the nuclear lncRNA signal.

Long non-coding RNA functions are still being determined, and many methods to help explore their roles rely on fresh tissue which is limited in supply. Formalin-fixed paraffin embedded (FFPE) tissue is used worldwide for tissue storage, preserves tissue architecture, and is kept indefinitely, so long-term clinical follow-up is possible. Thus, FFPE is a valuable resource for investigating not just lncRNA but other RNA. In addition, any cancer, not just breast cancer, can be studied using in situ hybridization probes for lncRNAs. RNA in situ hybridization of FFPE tissue offers a method to make observations on lncRNAs that may influence the cancer epigenome in a tissue-based technique.

### LncRNA and Polycomb Proteins

From prior studies [18], *HOTAIR* is known to recruit PcG proteins to chromatin through its interaction with the PRC2 complex, and, specifically, in the Gupta *et al*. paper [17], *HOTAIR* overexpression induces localization of PRC2 subunit EZH2 onto many genes in breast carcinoma. In this study, using in situ hybridization to evaluate *HOTAIR* lncRNA expression and immunohistochemistry to evaluate protein expression of EZH2, we confirm that EZH2 and *HOTAIR* are coexpressed in breast cancer, as they have the same expression score 75.7% of the time (*p*<0.0001). Thus, in breast carcinoma, there is increased *HOTAIR* expression and increased EZH2 expression. Although EZH2 did not have significant correlating expression scores with either nc*HoxA1* or nc*HoxD4*, *HOTAIR* and nc*HoxD4* did have the same score 68.4% of the time (*p*<0.0004). Lastly, nc*HoxA1* and nc*HoxD4* had the same expression score 82.6% of the time (*p*<0.0001). Correlating scores between these lncRNA in cancer suggest that, in some way, they may be acting in concert to modify the epigenome. To illustrate this idea, alone, nc*HoxA1* or nc*HoxD4* expression did not significantly correlate with proliferation index. However, when these two lncRNAs had correlating expression levels, the proliferation rate was more often increased (*p = *0.043), especially when they both had increased expression (*p = *0.052). Thus, nc*HoxA1* and nc*HoxD4* may act in parallel pathways or jointly to bring retarget PcG to genes.

### Clinicopathologic Correlations

The current study provides evidence that expression levels of lncRNA do trend with some clinicopathologic data, such that increased nc*HoxA1* trends with proliferation rate, and nc*HoxD4* trends with positive PR receptor status. In addition, the strong relationship between EZH2 and proliferation index in carcinoma is reconfirmed. However, it is interesting that strongly positive *HOTAIR* expression significantly correlated with ER and PR positivity, whereas strongly positive EZH2 significantly correlated with ER and PR negativity, even though *HOTAIR* and EZH2 had correlating expression levels. *HOTAIR* has been identified to interact with EZH2 and SUZ12 components of the PRC2 complex and to target the complex to silence transcription of genes at the *HOXD* locus [18]. In addition, *HOTAIR* has also been found to induce the localization of PRC2 subunits on 854 genes [17]. Thus, so far, the functions of *HOTAIR* depend on its interaction with PRC2. The PRC2 complex, however, is found to bind other lncRNAs besides *HOTAIR*; Khalil *et al*. [16] and Zhao *et al*. [23] identified thousands of lncRNAs, of which 24% associate with PRC2. Hence EZH2 in PRC2 has a much larger genomic range than that of *HOTAIR*. EZH2 has also been hypothesized to have neoplastic properties which are independent of the PRC2 complex [24]. In one such paper, Shi *et al*. [25] identified EZH2 to activate transcription in genes which are targets of estrogen and Wnt signaling pathways; this activity of EZH2 was not related to PRC2 complex activity. Thus, the broader outreach of EZH2 may allow it to better activate or influence genes involved in negative estrogen receptor expression breast carcinoma. *HOTAIR* may just be limited to its role in silencing the *HOXD* locus genes or the specific 854 genes, which could possibly lead to estrogen receptor positivity.

In this paper, we confirm that EZH2 expression correlated with ER and PR negativity. Unlike previous reports [8], we were able to find a significant association between the lack of HER2 overexpression (*p = *0.004) and triple negative breast carcinomas (*p = *0.0014). However, this finding is not too surprising, since EZH2 has been recently directly linked to BRCA1. In Gonzalez *et al*. [26], invasive ER-negative breast carcinomas showed EZH2 overexpression and BRCA1 downregulation. Downregulation of EZH2 expression in breast cancer cells led to decreased proliferation, delayed cell-cycle transition, and upregulation of the BRCA1 protein. In addition, a BRCA1 knockdown rescued the effects of EZH2. Since most *BRCA1*-related breast carcinomas (heritable mutations or sporadic defects) are frequently triple negative cancers (reviewed in Diaz *et al*. [27]) with BRCA1 protein downregulation, EZH2 is most likely upregulated in these carcinomas.

### Metastatic Breast Carcinoma and Survival

Though Gupta *et al*. [17] identified increased *HOTAIR* expression in a cross-sectional study of primary and metastatic breast carcinoma, and Kleer *et al*. [8] identified high levels of EZH2 in breast cancer metastases, this is the first study to look at matched primary and metastatic breast cancers and correlate EZH2 protein and *HOTAIR* expressions in archival material. In the first part of this study, neither *HOTAIR* expression nor EZH2 expression correlated with the clinicopathologic feature of metastases. However, upon comparing pairs of primary versus metastatic carcinoma, both *HOTAIR* and EZH2 expressions more often had equivalent (31% and 52%, respectively) or increased expression (52% and 40%, respectively) in the metastasis compared to the primary carcinoma. Thus, the data support increased expression of both EZH2 and *HOTAIR* in the metastatic cancers, compared to primary carcinoma.

Though the significance of the above data is limited by low numbers, a trend of increased EZH2 and *HOTAIR* expression in metastatic breast carcinomas is established. In addition, in primary carcinomas, strong expression of both EZH2 and *HOTAIR* also trends with worse survival (Figure 3). Of note, Gupta *et al*. [17] used quantitative PCR to demonstrate that elevated levels of *HOTAIR* in stage I and II breast cancer, defined as 125 fold more than that detected in normal breast epithelia, is strongly associated with eventual metastasis and death. The current study using RNA in situ hybridization of *HOTAIR* confirms this trend. The borderline logrank test for trend p-value of the Kaplan Meier curve (p = 0.0739) may be due to shorter clinical follow up of patients in the tissue microarray (thus limiting power to detect association), a low number of individuals with strong expression of both *HOTAIR* and EZH2, as well as less qualitative accuracy of ISH compared to qRT-PCR. However, the advantage of ISH is that it can be applied to FFPE tissue, and thus this study validates the concept of using lncRNA staining to evaluate cancer prognosis in parallel with protein–based biomarkers.

Overall, this paper confirms the link between lncRNAs, such as *HOTAIR*, and Polycomb Repressive Complex protein EZH2. Together, the lncRNAs and PRC2 may be influencing chromatin remodeling and the expression of other proteins to increase breast cancer invasiveness. These observations confirm the importance of understanding the chromatin phenotype in breast cancer, as chromatin remodeling and other such epigenetic changes are important in oncogenesis. By using RNA in situ hybridization, we have identified a way to investigate lncRNA expression in formalin-fixed paraffin embedded tissue. Using this tissue-based technique, the vast roles of lncRNAs can be further explored in multitudes of tissue and disease states.
